# Postoperative Complications Leading to Death after Coagulum Pyelolithotomy in a Tetraplegic Patient: Can We Prevent Prolonged Ileus, Recurrent Intestinal Obstruction due to Adhesions Requiring Laparotomies, Chest Infection Warranting Tracheostomy, and Mechanical Ventilation?

**DOI:** 10.1155/2013/682316

**Published:** 2013-02-28

**Authors:** Subramanian Vaidyanathan, Bakul Soni, Gurpreet Singh, Peter Hughes

**Affiliations:** ^1^Regional Spinal Injuries Centre, Southport and Formby District General Hospital, Town Lane, Southport PR8 6PN, UK; ^2^Department of Urology, Southport and Formby District General Hospital, Town Lane, Southport PR8 6PN, UK; ^3^Department of Radiology, Southport and Formby District General Hospital, Town Lane, Southport PR8 6PN, UK

## Abstract

A 22-year-old male sustained C-6 tetraplegia in 1992. In 1993, intravenous pyelography revealed normal kidneys. Suprapubic cystostomy was performed. He underwent open cystolithotomy in 2004 and 2008. In 2009, computed tomography revealed bilateral renal calculi. Coagulum pyelolithotomy of left kidney was performed. Pleura and peritoneum were opened. Peritoneum could not be closed. Following surgery, he developed pulmonary atelectasis; he required tracheostomy and mechanical ventilation. He did not tolerate nasogastric feeding. CT of abdomen revealed bilateral renal calculi and features of proximal small bowel obstruction. Laparotomy revealed small bowel obstruction due to dense inflammatory adhesions involving multiple small bowel loops which protruded through the defect in sigmoid mesocolon and fixed posteriorly over the area of previous intervention. All adhesions were divided. The wide defect in mesocolon was not closed. In 2010, this patient again developed vomiting and distension of abdomen. Laparotomy revealed multiple adhesions. He developed chest infection and required ventilatory support again. He developed pressure sores and depression. Later abdominal symptoms recurred. This patient's general condition deteriorated and he expired in 2011. *Conclusion*. Risk of postoperative complications could have been reduced if minimally invasive surgery had been performed instead of open surgery to remove stones from left kidney. Suprapubic cystostomy predisposed to repeated occurrence of stones in urinary bladder and kidneys. Spinal cord physicians should try to establish intermittent catheterisation regime in tetraplegic patients.

## 1. Background

Spinal cord injury patients are at increased risk for developing complications after surgery for renal stones. Symons and associates [1] reviewed twenty-nine patients with spinal neuropathy, who underwent percutaneous nephrolithotomy between October 1995 and January 2002. Thirty-nine percutaneous nephrolithotomy procedures were performed on 32 kidneys. There were two postoperative deaths. Major complications were associated with three procedures and consisted of seizures, aspiration pneumonia, and pressure necrosis. Symons and associates concluded that patients with spinal neuropathy and renal lithiasis posed a significant operative challenge. Technical difficulties and potential complications should be considered carefully before undertaking percutaneous nephrolithotomy in these patients. Culkin and associates [2] evaluated 23 male spinal cord injury patients (18 quadriplegic and 5 paraplegic patients), who underwent percutaneous nephrolithotomy. Major complications were associated with 4 of the 47 procedures (8.5 per cent) and consisted of a respiratory arrest, 2 perirenal abscesses, and a hydrothorax. Culkin and associates [2] concluded that the presence of infected stones prior operative procedures and medical complexity of these patients made complications more frequent.

We report a tetraplegic patient, who underwent coagulum pyelolithotomy of left kidney; this surgical procedure took six and a half hours. Dissection of perirenal tissue was very difficult and led to opening of pleura, peritoneum, and mesocolon. The rent in peritoneum could not be closed. Despite coagulum pyelolithotomy, complete clearance of renal calculi could not be achieved. This patient developed recurrent intestinal obstruction and underwent two laparotomies for division of adhesions.

The aim of this paper is to highlight the perils of open surgery for removal of stones from upper urinary tract in tetraplegic patients. We wish to draw special attention to delayed complications of suprapubic cystostomy in spinal cord injury patients. This tetraplegic patient had permanent suprapubic cystostomy and developed bilateral renal calculi and recurrent vesical calculi. Intermittent catheterisations along with antimuscarinic therapy instead of long-term suprapubic cystostomy would have greatly reduced the risk of renal and vesical calculi. Sadly, renal calculi contributed to the premature demise of this tetraplegic patient, a complication which could have been averted to a great extent by practising intermittent catheterisation regime rather than long-term indwelling urinary catheter drainage.

## 2. Case Presentation

A 22-year-old male patient fell down the stairs in 1992 at his girlfriend's house bumping his head but not losing consciousness. This person was immediately aware of his inability to move his arms or legs. Neurological examination revealed that he was C-6 complete tetraplegic. Initial management was conservative. This person spent first six weeks of his admission to spinal unit in bed on skull traction with six pounds weight. At the end of this period, there was no neurological recovery. Intravenous pyelography, performed in 1993, revealed that both kidneys and rest of the urinary tract were within normal limits. Urinary bladder was managed by four-hourly intermittent catheterisations, which were performed by his girlfriend. In 1993, the relationship with his girlfriend fell under strain. As this patient was unable to catheterise himself, a suprapubic catheter was inserted. In 2004, intravenous urography revealed opaque calculus upper pole calyx of left kidney, several large calculi in the bladder, and nondilated pelvicalyceal systems and ureters bilaterally. Open cystolithotomy was performed under spinal anaesthesia in 2004. This patient did well following surgery.

**Figure 1:**
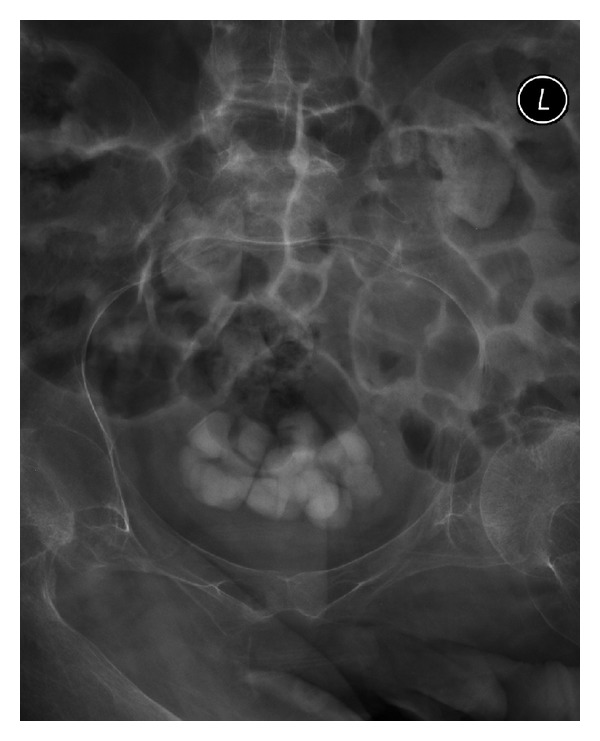
X-ray of abdomen, performed on 05 December 2008, revealed multiple calculi in urinary bladder.

In 2008, X-ray of abdomen revealed multiple bladder calculi and calculi in both kidneys (Figure 1). CT revealed large staghorn calculus lower pole of left kidney; moderate size staghorn calculus upper pole of right kidney; a large calculus in the proximal right ureter at the level of L-3; a large calculus in left ureter at L-3; and multiple large calculi in urinary bladder. Haemoglobin was 9.1 g/dL. Serum urea: 32.7 mmol/L; serum creatinine: 173 umol/L; glomerular filtration rate: 40 mL/minute. Open cystolithotomy was performed in 2008. Intrathecal block was attempted while the patient was lying in left lateral position. Bony resistance was noted in all directions at L3/4 and L4/5 intervertebral spaces. Therefore, this patient was intubated and received isoflurane anaesthesia. Postoperatively, haemoglobin was 8.4 g/dL. This patient received two units of blood.

**Figure 2:**
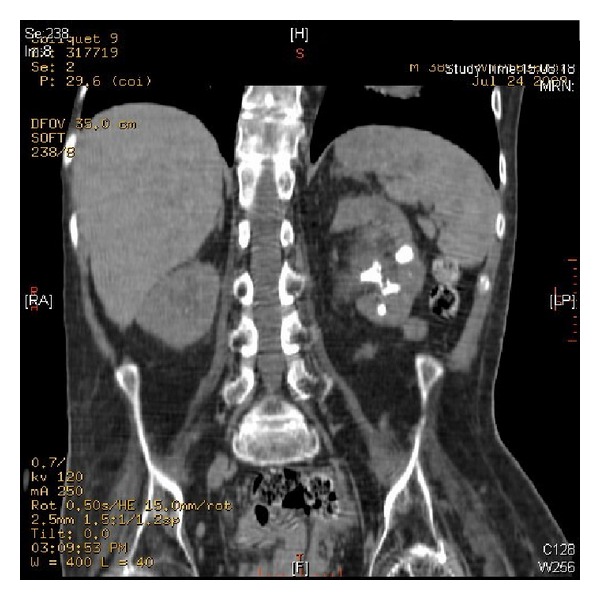
CT of urinary tract, performed on 24 July 2009, revealed multiple calculi in left kidney.

**Figure 3:**
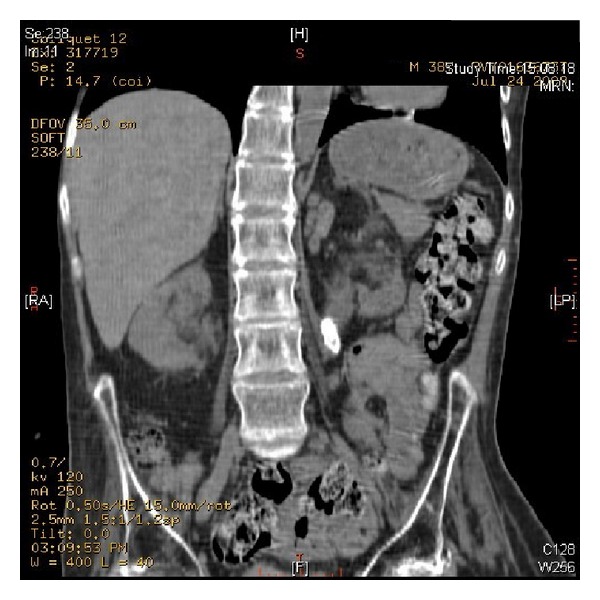
CT of urinary tract, performed on 24 July 2009, revealed two cm length calculus in left ureter at the level of L-3/4 causing mild hydronephrosis.

CT of urinary tract, performed in 2009, revealed multiple calculi in both kidneys (Figure 2). A 2 cm length calculus was seen in left ureter at the level of L-4 causing mild hydronephrosis (Figure 3). There was 1.5 cm length calculus in right pelviureteric junction causing mild hydronephrosis. Chest X-ray revealed clear lungs. In July 2009, open surgery was performed to remove stones from left kidney. The patient was positioned to lie on his right side. Twelfth rib was resected. Colon was distended. Exposure of left kidney was very difficult. Peritoneum and pleura were adherent. Left ureter was identified; very soft stone was removed. Coagulum pyelolithotomy was performed. Calcium chloride, thrombin, and cryoprecipitate were mixed and injected into renal pelvis. Coagulum was extracted. Chest drain was inserted. Peritoneum was not closed. Ureteric stent was kept. Duration of anaesthesia was 7 hours and 30 minutes. Surgery lasted for six and a half hours.

**Figure 4:**
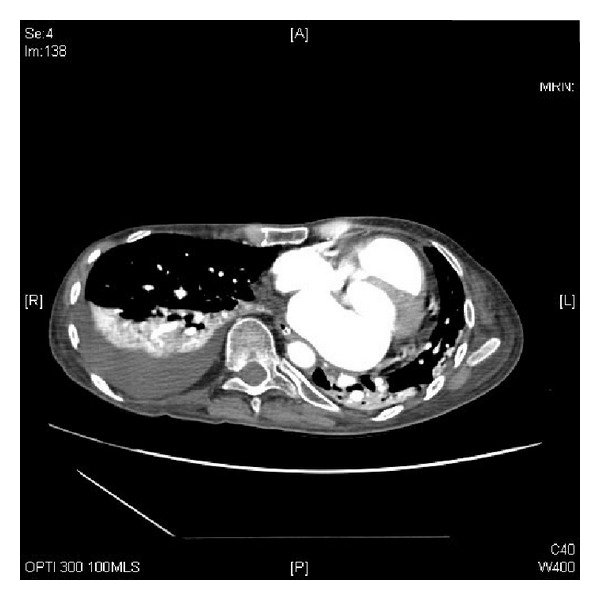
CT of chest, performed on 05 August 2009, revealed atelectasis and consolidation posteriorly in the right upper lobe and in both lower lobes. There was a moderately large right-sided pleural effusion.

Postoperatively, this patient developed tachycardia and hypotension. On fifth postoperative day, this patient developed tachypnoea, oxygen desaturation, and tachycardia of 170 per minute. He was intubated and ventilated. He required noradrenaline and adrenaline infusions in addition to propofol, fentanyl, and soluble insulin. CT pulmonary angiography revealed no pulmonary embolus. There was secondary atelectasis posteriorly in the upper right lobe and in both lower lobes. There was a moderate right-sided pleural effusion (Figure 4). The heart was displaced to the left side of midline due to scoliosis and depressed sternum.

**Figure 5:**
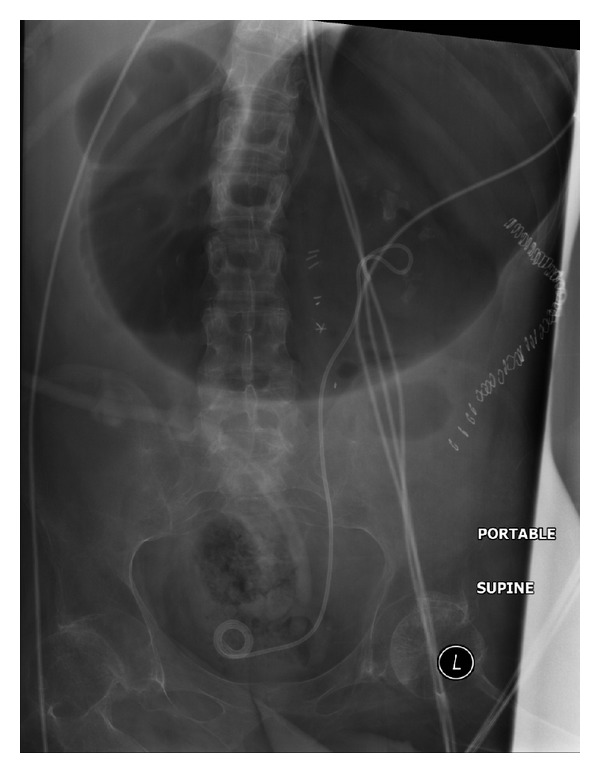
X-ray of abdomen, taken on 11 August 2009, revealed gastric distension.

Tracheostomy was performed on tenth postoperative day. He developed gastric distension (Figure 5). Total parenteral nutrition was continued, as he did not tolerate nasogastric feeding after renal surgery. He was prescribed erythromycin 250 mg every eight hours for the prokinetic effect.

**Figure 6:**
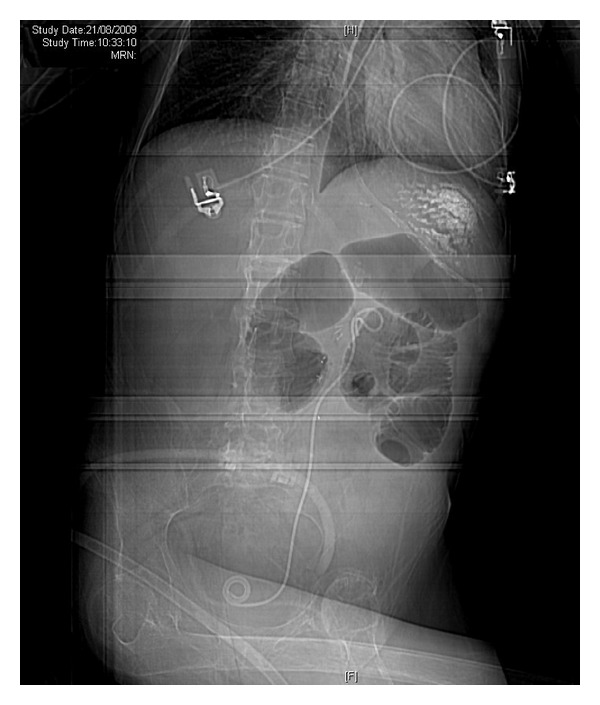
Scout film of abdomen, taken on 21 August 2009, showed dilated proximal small bowel loops.

**Figure 7:**
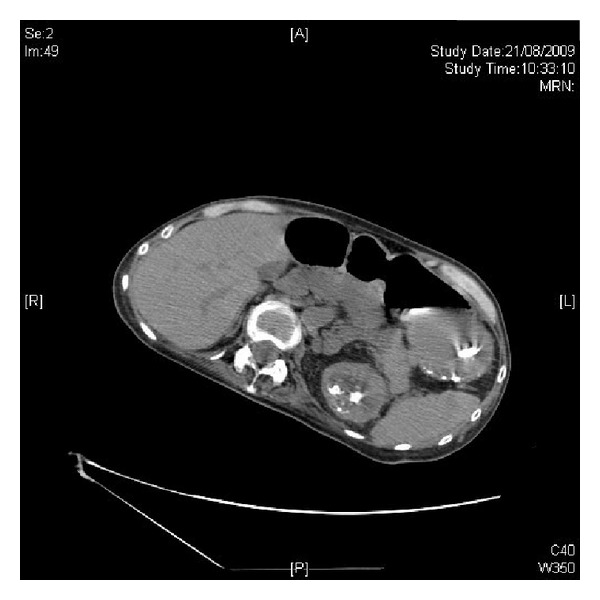
CT abdomen, performed on 21 August 2009, revealed multiple calculi in left kidney.

Three weeks after coagulum pyelolithotomy, CT of abdomen and pelvis was performed without intravenous contrast because of the patient's raised blood urea. There was atelectasis in the lingula. Despite a nasogastric tube *in situ*, the stomach was distended with air and fluid. The distension extended into proximal small bowel loops (Figure 6). The distal small bowel loops were not distended. Total parenteral nutrition was continued. Multiple calculi were seen in left kidney (Figure 7).

**Figure 8:**
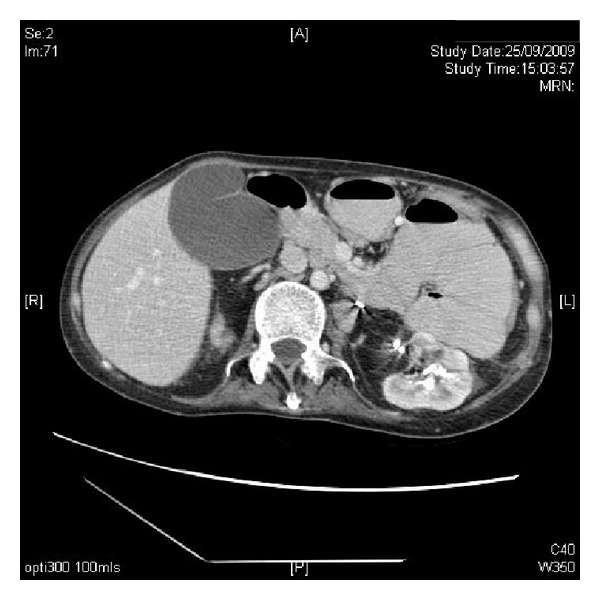
CT abdomen, performed on 25 September 2009, revealed loops of dilated proximal small bowel and distended gall bladder.

This patient still did not tolerate enteral feeding. Therefore, CT of abdomen was performed eight weeks after coagulum pyelolithotomy, and comparison was made with previous examination done five weeks earlier. Multiple calculi seen within both kidneys, with calculi noted in the left collecting system, pelvicalyceal system, and the proximal ureter. There was no evidence of significant hydronephrosis. There were still persistent loops of dilated proximal small bowel (Figure 8). There was oral contrast seen within the small bowel loops including the distal small bowel, suggestive of incomplete proximal small bowel obstruction with apparent transition zone in the distal jejunum. There appeared to be some rotation of the mesentery vessels, which could be secondary to adhesion or a band. There was distension of the gallbladder but no wall thickening or pericholecystic collection. No evidence of air within the gallbladder to suggest established infection. No focal liver lesion was noted. Pancreas and spleen appeared normal. There was no ascites. Right-sided pleural effusion with basal atelectasis was seen.

*Conclusion*. Evidence of proximal small bowel obstruction, most likely secondary to adhesional band: there was partial obstruction, as contrast was seen in distal small bowel.

The clinical and radiological features suggested mechanical postoperative obstruction, presumably due to adhesions, at the level of jejunum. Nine weeks after coagulum pyelolithotomy, laparotomy was performed for intestinal obstruction, as small bowel obstruction failed to resolve on conservative management. Mechanical small bowel obstruction was found at the level of mid-jejunum. The cause of obstruction was found to be dense inflammatory adhesions involving multiple small bowel loops protruding the defect in the sigmoid mesocolon and fixed posteriorly over the area of previous intervention and abdominal aorta. Several small bowel loops were attached posteriorly to the site of surgery on the left kidney. Dissection was difficult owing to inflammatory conditions. All adhesions were divided. The wide defect in the mesocolon was not closed. Three weeks later nasogastric feeding was commenced.

CT of urinary tract was performed in March 2010. Multiple bilateral opaque renal calculi were seen. Left ureteric J stent was *in situ*. There was no evidence of encrustations around the stent. Several calculi were seen in the right renal pelvis and at the pelviureteric junction. There was no opaque calculus in the line of the right ureter. There was a calculus in the posterior aspect of the bladder. Following this investigation, cystoscopy was performed. The ureteric stent was removed. Positioning for cystoscopy was very difficult because of fixed contractures of lower extremities. The patient's legs had to be held and supported.

In September 2010, this patient became unwell; he developed vomiting and distension of abdomen. Nasogastric tube was inserted and large amount of fluid was aspirated. CT abdomen suggested incomplete obstruction. As upper gastro-intestinal obstruction did not settle down, this patient was taken to theatre for lysis of adhesions and appendicectomy. Exploratory laparotomy was performed. Multiple adhesions were found. The level of obstruction was at the second or third part of duodenum. There was a partial obstruction to left kidney. After operation, it was difficult to wean the patient off the ventilator. While the patient was in intensive treatment unit, swelling and erythematic over right hip was noted. X-ray revealed subcapital fracture of neck of right femur, which was not present on previous CT scan. The fracture of femur was treated conservatively. This patient developed respiratory arrest; he was intubated. Subsequently, surgical tracheostomy was performed. This patient developed wound infection at the site of Hickman line. Microbiology revealed growth of methicillin-resistant *Staphylococcus aureus*, which was treated by vancomycin, administered intravenously.

In October 2010, this patient developed severe chest infection and was admitted to intensive care unit. He required ventilatory support. He developed pressure sores and depression. He refused basic care such as turning in bed. The hospital staff were concerned that they were not able to carry out basic nursing procedures. He was prescribed Citalopram 40 mg for depression.

In February 2011, this patient required hospital admission because of abdominal symptoms. Bilateral renal calculi were noted. Urologist decided not to do ureteric stents unless this patient developed symptoms related to urinary stones like sepsis or deteriorating renal function. This patient's general condition gradually deteriorated and he expired.

## 3. Discussion

The surgical management of urolithiasis has undergone a remarkable clinical evolution over the past three decades. The once common practice of open stone surgery has nearly been relegated to historical interest by the introduction of minimally invasive techniques, laparoscopy, and robot-assisted surgery [3]. The benefits of minimally invasive surgery to patients are reduced pain, shorter hospitalization, faster convalescence, improved cosmesis, and most importantly, reduced risk of abdominal as well as chest complications. Laparoscopic treatment is a viable option for large renal stone treatment with an excellent stone-free rate, especially when patients require their stones to be treated within a single session. However, laparoscopic techniques for removal calculi from urinary tract are more invasive than endourology procedures. Therefore, Kijvikai [4] cautioned that laparoscopic procedures should be reserved as the last resort option for renal stone management in the modern endourology era. This patient underwent open surgery after excising twelfth rib to remove stones from left kidney and developed life-threatening complications. Zacchero and associates [5] recommended laparoscopic transmesenteric approach as it represented an advantageous technical improvement of minimally invasive surgery for treatment of left renal diseases.

Following coagulum pyelolithotomy, our patient developed recurrent intestinal obstruction. Small bowel obstruction has been one of the most common emergent complications of abdominal surgery; intra-abdominal adhesions are the leading cause of small bowel obstruction. Postoperative small bowel obstruction occurred in 131 of 1,910 children (6.9%) enrolled in the Third National Wilms' Tumor Study [6]. The aetiology of small bowel obstruction was bowel adhesions in 104 cases, intussusceptions in 17, internal hernia in 2, and uncertain in the remaining 8 children.

Peritoneal adhesions have been reported as the cause of 32% of acute intestinal obstruction and 65–75% of all small bowel obstructions. It is estimated that peritoneal adhesions develop after 93–100% of upper abdominal laparotomies and after 67–93% of lower abdominal laparotomies, but only 15–18% of these adhesions require surgical reintervention [7]. The laparoscopic approach appears to decrease the risk of adhesion formation by 45% and the need for adhesion-related reintervention to 0.8% after appendectomy and to 2.5% after colorectal surgery.

In recent years, a common strategy for the prevention of postsurgical intra-abdominal adhesions has been intrasurgical placement of adhesion barriers into the peritoneal cavity. Osmotic agents, such as various polysaccharides, frequently are used as antiadhesive materials. A recent Cochrane review [8] found the absorbable adhesion barrier, Interceed to reduce the incidence of adhesion formation following laparoscopy and laparotomy. Gore-Tex might be superior to Interceed in preventing adhesion formation, but its usefulness was limited by the need for suturing and later removal. There was no evidence of effectiveness of Seprafilm and Fibrin sheet in preventing adhesion formation. However, Hashimoto and associates [9] found the hyaluronate carboxymethyl cellulose-based bioresorbable membrane (HC membrane; Seprafilm) to effectively reduce the severity of wound adhesion. Cohen and associates [10] also showed glycerol hyaluronate/carboxy-methyl cellulose to effectively reduce adhesions to the midline incision and adhesions between the omentum and small bowel after abdominal surgery. However, it should be emphasised that adverse events were similar between treatment and no treatment control groups with the exception of abscess and incisional wound complications, which were more frequently observed with glycerol hyaluronate carboxymethyl cellulose.

A systematic review of the English and French language surgical literature published between 1995 and 2009 was performed by Ouaïssi and associates [7] using the keywords “adhesion” and “surgery.” These authors found that at the present time, only one product consisting of hyaluronic acid applied to a layer of carboxymethyl cellulose (Seprafilm) had been shown to significantly reduce the incidence of postoperative adhesion formation; but this product was also associated with a significant increase in the incidence of anastomotic leakage when the membrane was applied in direct contact with the anastomosis. The use of this product had not been shown to decrease the risk of reintervention for bowel obstruction.

Osmotic agents, such as various polysaccharides, are frequently used as antiadhesive materials and these may affect kidney function. Economidou and associates [11] reported a case of an individual with preexisting chronic kidney disease who developed acute kidney injury after surgical placement of an antiadhesive barrier of macromolecular polysaccharides. A kidney biopsy, performed because of persistent kidney failure, showed tubular cell lesions compatible with osmotic nephrosis lesions. This case suggested that the use of polysaccharide-containing antiadhesive barriers could induce severe kidney damage and, therefore, such barriers should be used with caution in patients with abnormal kidney function.

Coagulum pyelolithotomy has been associated with a few serious complications albeit rarely. Pence II and associates [12] reported an operative death due to a pulmonary embolus. McVary and O'Conor [13] reported a case of transmission of viral hepatitis during coagulum pyelolithotomy. In our patient, perirenal dissection proved to be very difficult, and peritoneal cavity had been opened. It is possible that spillage of even small amount of coagulum components could have contributed to formation of adhesions between intestines and renal bed.

## 4. Conclusions

- A tetraplegic patient developed recurrent intestinal obstruction, pulmonary consolidation, and respiratory failure following coagulum pyelolithotomy. Risk of these complications could have been reduced if minimally invasive surgery had been performed instead of open surgery to remove stones from left kidney.
- This tetraplegic patient, who had been managing his bladder by permanent suprapubic cystostomy, developed bilateral renal calculi and recurrent vesical calculi. Intermittent catheterisations along with antimuscarinic therapy instead of long-term suprapubic cystostomy would have greatly minimised the risk of renal and vesical calculi. This patient's girlfriend performed intermittent catheterisations soon after he sustained tetraplegia. When their relationship broke down, nobody was available to carry out intermittent catheterisations and suprapubic cystostomy was performed. Although suprapubic cystostomy solved the immediate problem, in the long run, the indwelling urinary catheter predisposed to repeated occurrence of stones in urinary bladder and kidneys. Spinal cord physicians should leave no stones unturned to establish intermittent catheterisation regime, particularly in tetraplegic patients.
- In this patient, peritoneum was opened during dissection of perirenal tissue and there was a rent in mesocolon, which could not be closed. It is debatable whether the use of hyaluronate carboxymethyl-cellulose-based bioresorbable membrane might have diminished the risks of adhesions and need for repeated laparotomies.
- It is questionable whether even minute spillage of coagulum components during surgery when peritoneum was open could have contributed to formation of adhesions and produced intestinal obstruction.
